# Serum Uromodulin Levels in Prediction of Acute Kidney Injury in the Early Phase of Acute Pancreatitis

**DOI:** 10.3390/molecules22060988

**Published:** 2017-06-14

**Authors:** Beata Kuśnierz-Cabala, Agnieszka Gala-Błądzińska, Małgorzata Mazur-Laskowska, Paulina Dumnicka, Mateusz Sporek, Aleksandra Matuszyk, Krzysztof Gil, Piotr Ceranowicz, Jerzy Walocha, Jakub Kucharz, Michał Pędziwiatr, Krzysztof Bartuś, Rafał Trąbka, Marek Kuźniewski

**Affiliations:** 1 Department of Diagnostics, Chair of Clinical Biochemistry, Jagiellonian University Medical College, 31-501 Kraków, Poland; mbkusnie@cyf-kr.edu.pl; 2 St. Queen Jadwiga Clinical District Hospital No2 in Rzeszow, 35-301 Rzeszów, Poland; agala.edu@gmail.com; 3 Department of Diagnostics, University Hospital, 31-501 Kraków, Poland; mbmazur@cyf-kr.edu.pl; 4 Department of Medical Diagnostics, Jagiellonian University Medical College, 30-688 Kraków, Poland; paulina.dumnicka@uj.edu.pl; 5 Department of Anatomy, Jagiellonian University Medical College, 31-034 Kraków, Poland; msporek1983@gmail.com (M.S.); aleksandra.matuszyk@uj.edu.pl (A.M.); jwalocha@cm-uj.krakow.pl (J.W.); 6 Surgery Department, The District Hospital, 34-200 Sucha Beskidzka, Poland; 7 Department of Pathophysiology, Faculty of Medicine, Jagiellonian University Medical College, 31-121 Kraków, Poland; mpgil@cyf-kr.edu.pl; 8 Department of Physiology, Jagiellonian University Medical College, 31-531 Kraków, Poland; 9 Department of Experimental and Clinical Surgery, Jagiellonian University Medical College, 31-126 Kraków, Poland; jakub.kucharz@uj.edu.pl; 10 2nd Department of Surgery, Faculty of Medicine, Jagiellonian University Medical College, 31-501 Kraków, Poland; michal.pedziwiatr@uj.edu.pl; 11 Department of Cardiovascular Surgery and Transplantology, Faculty of Medicine, Jagiellonian University Medical College, 31-202 Kraków, Poland; krzysztof.bartus@uj.edu.pl (K.B.); rafal.trabka@uj.edu.pl (R.T.); 12 Chair and Department of Nephrology, Jagiellonian University Medical College, 31-501 Kraków, Poland; marek.kuzniewski@uj.edu.pl

**Keywords:** glycoproteins, uromodulin, acute pancreatitis, acute kidney injury

## Abstract

In health, uromodulin is the main protein of urine. Serum uromodulin concentrations (sUMOD) have been shown to correlate with kidney function. Acute kidney injury (AKI) is among the main complications of severe acute pancreatitis (AP). No reports exist on sUMOD in patients with AP, including the diagnostic usefulness for early prediction of AP severity. We measured sUMOD during first 72 h of AP. Sixty-six adult patients with AP were recruited at the surgical ward of the District Hospital in Sucha Beskidzka, Poland. AP was diagnosed according to the Revised Atlanta Classification. Blood samples were collected at 24, 48 and 72 h of AP, and sUMOD concentrations were measured with enzyme-linked immunosorbent test. sUMOD decreased non-significantly during the study. Patients with severe AP had non-significantly lower sUMOD concentrations than those with mild disease. Significant positive correlation was observed between sUMOD and estimated glomerular filtration rate on each day of the study and negative correlations were shown between sUMOD and age, serum creatinine, cystatin C and urea. Patients with AKI tended to have lower sUMOD. Although sUMOD correlated significantly with kidney function in the early phase of AP, measuring sUMOD did not allow to reliably predict AP severity or development of AKI.

## 1. Introduction

Prevalence of acute pancreatitis (AP) equals approximately 10–100 cases per 100,000 people per year. Mortality rates may reach 30%, despite progress in understanding the pathogenesis and implementation of intensive care in patients with moderately severe AP (MSAP) and severe AP (SAP) [1,2,3]. AP is related to premature activation of pancreatic proenzymes causing damage to pancreas and adjacent tissues, leading to strong inflammatory response. In part of the patients, systemic complications develop, including vascular leak syndrome and acute kidney injury (AKI). AKI occurs in nearly 15% of patients with AP and is associated with high mortality, reaching over 80% [2,3]. In older patients (above 60 years of age), the mortality rates in AP increase significantly [4]. Early prediction of AP severity and selection of potentially complicated cases is crucial to appropriately address the intensive care. Currently, the prediction of AP severity is based on clinical assessment of circulatory, respiratory and renal efficiency (Modified Marshall Scoring System), and the signs of systemic inflammatory response syndrome (SIRS). The assessment includes routine laboratory tests such as serum creatinine, urea and C-reactive protein (CRP), blood leukocyte count, or arterial blood gases [5,6,7]. In practice, early prognosis of AP severity is not an easy task. Currently, the development of transient or persistent organ failure is considered the main determinant of AP severity [7]. Early assessment of severity, followed by intensive monitoring and treatment in severe cases is currently the most effective measure to reduce mortality from AP [2,8,9,10]. New biomarkers are sought that would allow faster and more reliable prognosis of AP severity, and ideally, also the monitoring of the course of the disease and the effectiveness of treatment. In particular, the markers that would allow earlier diagnosis of AKI as compared to serum creatinine are highly awaited. In our earlier studies, we have shown that the markers of endothelial dysfunction and increased vascular leak (angiopoietin-2 and soluble fms-like tyrosine kinase-1) as well as the markers of kidney injury (neutrophil gelatinase-associated lipokalin in urine and in serum) are significantly associated with acute kidney injury in the early phase of AP as well as with subsequent severe course of AP [11,12,13,14].

Uromodulin (UMOD), also known as Tamm-Horsfall glycoprotein is the main protein that occurs physiologically in urine. Healthy adults secrete daily between 100 and 200 mg of this protein in urine [15]. UMOD is a glycoprotein with molecular weight of 80–90 kDa, and is expressed within the ascending limb of the loop of Henle and the opening part of distal renal tubule [16]. Small concentrations of UMOD are also present in serum (sUMOD). The biological role of sUMOD has not been elucidated, however, decreased sUMOD has been shown to strongly correlate with deteriorating kidney function in chronic kidney disease [17,18,19,20]. Importantly, sUMOD is a tubular marker in contrast to routinely used markers of glomerular filtration such as serum creatinine or serum cystatin C [21]. Moreover, as a marker of renal failure, sUMOD seems to possess several important advantages over serum creatinine. UMOD is exclusively produced in renal tubules, therefore its serum concentrations reflect renal tubular mass, and are not influenced by muscle mass or diet [19,20]. Moreover, sUMOD has been shown to decrease early in the course of chronic kidney disease, making it possible to identify patients with stage 1 of the disease [17]. Until now, sUMOD has not been studied in the context of AKI, although several studies have explored urine concentrations of UMOD in assessment of AKI among patients with cardiovascular events [22] as well as in newborns and fetuses [23,24]. The diagnostic utility of measuring sUMOD levels in patients with AP, including those with AKI in the course of AP, has not been determined.

The aim of the study was to assess the concentrations of sUMOD in the early phase of AP (first 72 h of the disease) and to evaluate the utility of sUMOD for the early prediction of AP severity, including the development of AKI in the course of the disease.

## 2. Results

Sixty-six patients were recruited for the study. The group included 32 (48%) women and 34 (52%) men in the average age of 61 ± 18 years, range 23–86 years. Biliary etiology was most common (n = 34; 52%), followed by alcohol (n = 18; 27%), and hypertriglyceridemia (n = 5; 8%). One patient (2%) developed AP as a complication of endoscopic retrograde cholangiopancreatography. Other etiology or idiopathic AP was diagnosed in eight patients (12%). At admission, most patients were diagnosed with comorbid conditions, including hypertension (n = 22; 33%), coronary artery disease (n = 18; 27%), diabetes mellitus (n = 10; 15%), pulmonary diseases (n = 7; 11%), and chronic kidney disease (n = 3; 5%).

In the studied group, local complications were observed in eight patients (12%). Thirteen patients (20%) developed pleural effusion. Transient organ failure was observed in six patients (9%) and persistent organ failure in 5 (8%). Mild AP (MAP) was diagnosed in 46 (70%) of the patients, MSAP in 15 (23%), and SAP in five patients (8%) (Table 1). Three patients (5%) required surgical intervention in the course of AP. Eleven patients (17%) were diagnosed with AKI.

As expected, more severe AP was associated with significantly longer hospital stay and higher bedside index of severity in AP (BISAP) scores (Table 1). At admission, patients with more severe AP presented with higher concentrations of the markers of renal function (i.e., urea, creatinine and cystatin C), and, consequently, with lower estimated glomerular filtration rates (eGFR) (Table 1). Moreover, more severe AP was associated with higher serum C-reactive protein (CRP). Lower serum albumin and lower lymphocyte counts were observed among patients with SAP (Table 1).

**Figure 1:**
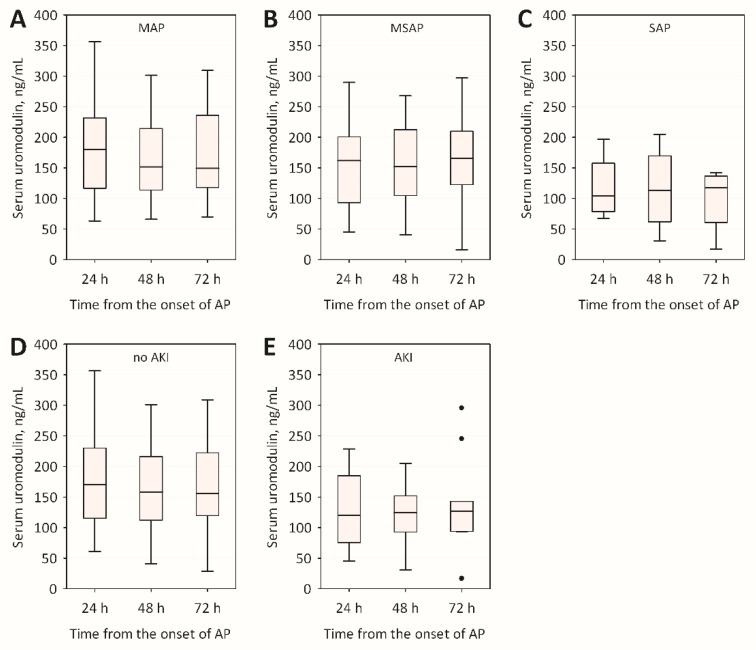
Changes in serum uromodulin concentrations during the first 72 h of acute pancreatitis among patients with: mild acute pancreatitis (MAP) (**A**); moderately-severe acute pancreatitis (MSAP) (**B**); and severe acute pancreatitis (SAP) (**C**); as well as among patients: without acute kidney injury (AKI) (**D**); and with AKI (**E**).

**Figure 2:**
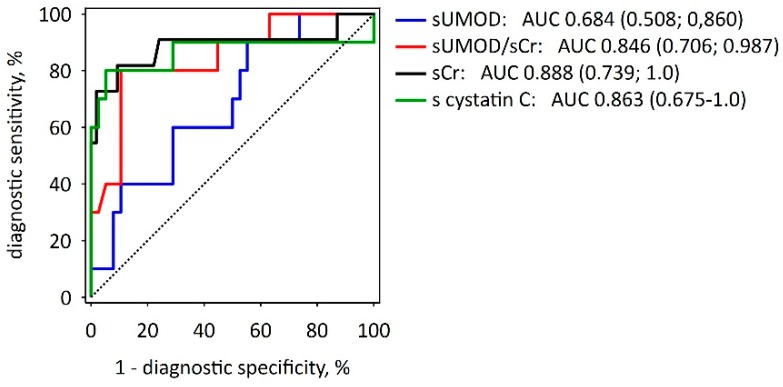
Receiver operating characteristic curves showing the diagnostic utility of selected serum markers measured at 24 h from the onset of AP symptoms in prediction of AKI in the course of AP. The values of area under curve (AUC) with 95% confidence intervals (in brackets) are reported for serum uromodulin (sUMOD), sUMOD/serum creatinine ratio (sUMOD/sCr), serum creatinine alone (sCr), and serum cystatin C (s cystatin C). The diagonal dotted line is the line of no-discrimination.

Average serum uromodulin concentrations during the first 72 h from the onset of AP symptoms were consistently lower in SAP patients as compared to MAP or MSAP, although the differences were not statistically significant (Table 1, Figure 1A–C). In addition, patients with AKI had on average lower sUMOD comparing to those without AKI, but the differences were not statistically significant (Figure 1D,E). At admission, neither sUMOD, nor sUMOD/serum creatinine ratio provided better prediction of AKI than serum creatinine alone or serum cystatin C (Figure 2). There were no significant trends towards the increase or decrease in sUMOD concentrations with time during the first 72 h of AP course (Figure 1).

Serum uromodulin concentrations measured during first 48 h of AP were negatively associated with age (Table 2). At admission, sUMOD negatively correlated with BISAP score, CRP and neutrophil counts (Table 2). Of note, significant correlations were observed between sUMOD and the markers of renal function during the entire study (Table 2). Serum uromodulin correlated negatively with serum creatinine, cystatin C and urea and positively with eGFR values. The association between sUMOD and eGFR was independent of age and sex during the entire study, despite strong correlation between age and eGFR (R = −0.77; *p* < 0.001): the results of multiple linear regression are shown in Table 3.

## 3. Discussion

Most of available reports on sUMOD regard chronic kidney disease [17,18,19,20]. Our study is the first to assess sUMOD concentrations in patients with the early phase of AP of various severity, including patients with AKI. The diagnostic utility of sUMOD in the prognosis of AP severity has not been evaluated before. In our study, sUMOD concentrations did not allow reliable prediction of AP severity during the first 72 h from the onset of AP symptoms. However, in the early phase of AP, sUMOD correlated significantly with currently used markers of renal function: positively with eGFR and negatively with serum creatinine, cystatin C and urea. These correlations confirm the interesting property of sUMOD: high concentrations are associated with good renal function while low levels indicate deteriorating renal function. In the recent study of Steubl et al. [17], plasma UMOD concentrations of 167.6 ± 53.6 ng/mL were observed among individuals without kidney disease, while the concentrations decreased with increasing stage of chronic kidney disease. In our study, sUMOD levels among patients with MAP and without AKI were similar to those observed by others among people with normal renal function [17,18]. Our observations are also in line with previous reports showing positive relationship between sUMOD concentrations and eGFR [17,19,20,25]. However, the strength of this correlation is lower in our study (including patients with AKI) comparing to the studies including patients with chronic kidney disease [17,19,20].

In our study, sUMOD negatively correlated with patient’s age. Physiologically, renal function deteriorates with age, as indicated by an increase in serum creatinine and cystatin C concentrations and a decrease in GFR in older population. This observation is consistent with previous studies [17,19,20]. In addition, urine UMOD excretion has been shown to decrease in older patients, reflecting decreasing tubular mass [21,26].

In chronic kidney disease, serum UMOD may serve as a new indicator of renal function with a diagnostic value comparable (or complementary) to eGFR [17,19,20]. However, our results indicate lower diagnostic utility of sUMOD in AKI as compared to what has been shown in chronic kidney disease [17,20]. In AP patients, sUMOD showed lower diagnostic utility for early recognition of AKI as compared to serum creatinine and cystatin C. In previous studies, low preoperative urine UMOD to urine creatinine ratios were shown to significantly predict AKI after cardiac surgery [22], while urine concentrations of UMOD have been reported to be decreased among infants with AKI [23]. These results may be in part explained by higher vulnerability to AKI among subjects with lower kidney mass (including lower tubular mass). However, in vitro studies have shown decreased expression of UMOD following kidney ischemia-reperfusion injury [27,28]. Recent study of El-Achkar et al. [29] showed low tubular expression of UMOD in mice at peak injury after kidney ischemia-reperfusion, following by increased UMOD expression during recovery, with translocation to basolateral part of tubular cells and increased serum concentrations of UMOD. This would suggest dynamically changing sUMOD concentrations during AKI, depending on time from initial injury. We were not able to identify previous studies on sUMOD concentrations in human patients with AKI. In our patients with AP (including the subgroup with AKI), no significant time related changes in sUMOD were observed, however, this may reflect variable dynamics of AKI in our patients.

UMOD is anchored to the cell membrane of the epithelial cells of the ascending limb of the loop of Henle with a glycosylphosphatidylinositol anchor located in the apical part of the tubular epithelial cells [16,30,31]. Following enzymatic cleavage, UMOD is shed into the lumen of the renal tubule. Smaller quantities of UMOD are also expressed in the basolateral membrane of tubular cells [32]. This way, the protein reaches the renal interstitial space and the blood. Serum concentrations of UMOD in healthy humans range from 70 to 540 ng/mL [33]. The significance and mechanism of UMOD expression in the basolateral area of the renal tubular cells have not been explained so far [34]; however, it has been associated both with stimulating the pro-inflammatory response and intensifying kidney injury [35] and with the anti-inflammatory activity [36]. Based on the present understanding, which defines UMOD as a kidney-specific protein (produced solely by renal tubular cells), it seems especially worth studying the presumed active response of kidneys to the developing systemic inflammation associated with the early phase of severe AP. In vitro studies have demonstrated that UMOD is a strong activating factor for human granulocytes, both in UMOD-incubated granulocyte colonies grown from peripheral blood and in colonies of the renal tubular epithelial cells, in which an increased production of UMOD was detected in their apical part, with a simultaneous increased activity of granulocytes incubated with a supernatant of the UMOD-producing epithelial tubular cells [37]. UMOD has been shown to be a chemoattractant for monocytes and neutrophils [37]. Moreover, Wimmer et al. [38] demonstrated that the effectiveness of chemotaxis on inflammatory cells in vitro depends on UMOD concentrations: low concentrations exerted stimulating effect, while high concentrations had inhibitory effect on chemotaxis of leukocytes and apoptosis. Low concentrations of UMOD increase neutrophils’ migration towards the inflammatory focus, while with higher UMOD concentrations, a process of cellular inactivation occurs at the site of developing inflammation [39]. In our study, weak negative correlations were observed between sUMOD and blood neutrophil count as well as serum CRP concentrations.

The main limitation of our study is limited number of patients included. However, considering the previously reported sUMOD concentrations among subjects with normal kidney function of 167.6 ± 53.6 ng/mL [16], we should be able to detect 1-standard deviation difference between AKI and non-AKI patients with the power of 84%. Of note, in the studied group, serum creatinine, cystatin C and eGFR differed significantly both between AKI and non-AKI patients and between patients with MAP and SAP. Therefore, our preliminary findings do not support better diagnostic utility of sUMOD in AKI as compared to traditional markers of renal impairment. Still, this conclusion must be limited to AKI in the course of AP, and should be verified by larger studies.

In summary, our study is the first report regarding sUMOD concentrations in the course of AP complicated with AKI. In the early phase of AP, low sUMOD indicates deteriorating renal function. However, sUMOD measurements during the first 72 h from the onset of AP symptoms do not allow better prediction of the severity of AP or the development of AKI in the course of AP as compared to currently used markers (including serum creatinine).

## 4. Materials and Methods

### 4.1. Patients and Study Protocol

Consecutive adult patients hospitalized and treated for AP in the surgical ward of the District Hospital in Sucha Beskidzka, Poland were recruited for the study. The study included patients who were admitted within the first 24 h from the onset of AP symptoms. All patients provided written informed consent for participation in the study. The research project was approved by the Bioethical Committee of the Jagiellonian University (No of approval 122.6120.241.2015).

AP was diagnosed according to the current Revised Atlanta Classification 2012 [7], i.e., when at least two out of the three following criteria were fulfilled: the epigastric pain typical of AP, at least a threefold increase in activity of pancreatic enzyme (amylase or lipase) and the relevant findings in imaging examinations (computed tomography with contrast enhancement, magnetic resonance imaging or transabdominal ultrasonography) [7,10]. The disease severity was classified according to the Revised Atlanta Classification [7] based on the course of AP during the hospital stay. The group with mild AP (MAP) included patients in whom neither organ failure, nor local or systemic complications were observed. Moderately severe AP (MSAP) was diagnosed in patients with temporary organ failure (lasting up to 48 h), local complications (acute peripancreatic fluid collection, pancreatic pseudocyst, acute necrotic collection and walled-off necrosis) and/or exacerbation of preexisting comorbidities. In case of persistent organ failure (lasting over 48 h), the patient was qualified to the group with severe AP (SAP).

Acute kidney injury was diagnosed according to Kidney Disease: Improving Global Outcomes (KDIGO) initiative guidelines, i.e., when serum creatinine increased at least 1.5-times within 7 days or at least by 26.5 µmol/L from baseline within 48 h, or urine output was <0.5 mL/kg/h for at least 6 h [23].

### 4.2. Laboratory Measurements

Blood samples for laboratory tests were drawn from the patients thrice: at admission (i.e., within first 24 h from the onset of symptoms), on Day 2 (about 48 h from the onset of symptoms) and on Day 3 of AP (about 72 h from the onset of symptoms). Routine laboratory tests used for an ongoing assessment and monitoring of patients were performed in the Laboratory Diagnostic Department of the District Hospital in Sucha Beskidzka, Poland, on the day of order. Glomerular filtration rate (eGFR) was estimated based on serum creatinine and cystatin C concentrations according to Chronic Kidney Diseases Epidemiology Collaboration (CKD-EPI) equation [24].

Serum samples for uromodulin measurements were aliquoted and stored at −80 °C until assayed with enzyme-linked immunosorbent test. Human Uromodulin ELISA kits (BioVendor, Brno, Czech Republic) were used. The readings were made with an automatic microplate reader Automatic Micro ELISA Reader ELX 808 (BIO-TEK^®^ Instruments Inc., Winooski, VT, USA). The reference values determined by the manufacturer of the kit were between 37.0 and 501.0 ng/mL. The limit of detection was 0.12 ng/mL. sUMOD measurements were conducted in the Diagnostic Department, Chair of Clinical Biochemistry, Jagiellonian University, Krakow, Poland.

BISAP score was calculated based on the clinical and laboratory data obtained during first 24 h from the onset of AP symptoms [4].

### 4.3. Statistical Analysis

Data are reported as number (percentage of the respective group) for categories, mean ± standard deviation for normally distributed quantitative variables and median (lower-upper quartile) for non-normally distributed quantitative variables. Distributions were tested for normality with Shapiro–Wilk’s test. Differences between groups were tested with chi-squared test, one-way ANOVA or Kruskal–Wallis ANOVA, respectively. Spearman correlation coefficients are reported for simple correlations. Multiple linear regression was calculated in order to check whether serum uromodulin correlates with other biomarkers of kidney function independently of the age of patients; right-skewed variables were log-transformed before the analysis. Receiver operating characteristic curves were used to present diagnostic utility; values of area under curve (AUC) with 95% confidence intervals were reported. All the tests were two-tailed. The results were considered significant at *p* ≤ 0.05. Statistica 12.0 (StatSoft, Tulsa, Oklahoma, USA) was used for computations.

## Figures and Tables

**Table 1:** Clinical characteristics of patients with acute pancreatitis and the results of laboratory tests at admission with respect to the severity of acute pancreatitis.

Variable	MAP (n = 46)	MSAP (n = 15)	SAP (n = 5)	*p* *
Age, years	59 ± 19	64 ± 16	70 ± 19	NS
Male gender, n (%)	25 (54)	6 (40)	3 (60)	NS
Duration of hospital stay, days	6 (5–7)	12 (10–17)	27 (13–31)	<0.001 ^a,b^
BISAP score ≥3 in first 24 h, n (%)	0	2 (13)	4 (80)	<0.001 ^d^
Comorbidities, n (%)	33 (72)	12 (80)	5 (100)	NS
Mortality, n (%)	0	0	3 (60)	-
Amylase, U/L	1085 (571–1722)	1038 (772–1917)	1013 (357–1909)	NS
Urea, mmol/L	5.5 (4.1–6.8)	6.7 (5.6–8.4)	13.4 (11.7–15.1)	0.002 ^a^
Creatinine, µmol/L	72.7 (63.4–94.8)	90.7 (67.6–113.4)	194.4 (120.0–228.0)	0.008 ^a^
Cystatin C, mg/L	0.87 (0.65–1.07)	0.84 (0.76–1.58)	1.98 (1.65–2.42)	0.009 ^a^
eGFR, mL/min/1.73 m^2^	84.1 (61.2–120.7)	71.5 (41.7–116.6)	29.0 (24.7–33.7)	0.005 ^a^
Uromodulin, ng/mL	180 (116–232)	162 (93–201)	104 (79–158)	NS
CRP, mg/L	5.9 (1.9–48.6)	24.4 (9.0–103.2)	191.1 (74.6–258.2)	0.003 ^a^
Albumin, g/dL	40.6 ± 4.0	39.7 ± 3.9	30.7 ± 8.4	0.030 ^a^
WBC, ×10^3^/µL	11.1 (9.2–14.6)	12.4 (10.6–15.3)	10.4 (9.8–18.4)	NS
MONO, ×10/µL	0.51 (0.40–0.73)	0.51 (0.26–0.76)	0.43 (0.25–0.46)	NS
NEU, ×10^3^/µL	9.18 (7.35–12.71)	9.38 (6.59–14.06)	9.90 (8.78–15.43)	NS
LYM, ×10^3^/µL	1.31 (0.95–1.80)	1.63 (1.17–2.87)	0.57 (0.36–1.01)	0.006 ^a,c^
Hematocrit, %	42.5 ± 4.1	43.2 ± 6.7	39.0 ± 7.1	NS
PLT, ×10^3^/µL	237 (196–255)	230 (210–267)	128 (121–150)	NS

* *p*-value for overall difference between the three groups; superscript letters denote significant differences in post-hoc tests: ^a^ between MAP and SAP; ^b^ between MAP and MSAP; ^c^ between MSAP and SAP; ^d^ in all between-groups comparisons. Abbreviations: MAP, mild acute pancreatitis; MSAP, moderately-severe acute pancreatitis; SAP, severe acute pancreatitis; NS, non-significant; BISAP, bedside index of severity in acute pancreatitis; eGFR, estimated glomerular filtration rate; CRP, C-reactive protein; WBC, white blood cell count; MONO, monocyte count; NEU, neutrophil count; LYM, lymphocyte count; PLT, platelet count.

**Table 2:** Correlations between serum uromodulin concentrations and the selected variables (including the biomarkers of renal function) during the first 72 h of acute pancreatitis.

Variable	24 h		48 h		72 h	
	R	*p*	R	*p*	R	*p*
Age	−0.43	0.002	−0.28	0.049	−0.22	NS
BISAP *	−0.36	0.011	-	-	-	-
CRP	−0.29	0.043	−0.11	NS	−0.13	NS
NEU	−0.29	0.044	−0.10	NS	−0.07	NS
Creatinine	−0.47	<0.001	−0.46	0.001	−0.34	0.015
eGFR	0.47	<0.001	0.49	<0.001	0.38	0.009
Cystatin C	−0.48	<0.001	−0.51	<0.001	−0.45	0.002
Urea	−0.51	<0.001	−0.44	0.001	−0.37	0.010

* Bedside index of severity in acute pancreatitis (BISAP) score was only assessed on the first day of hospital stay (first 24 h of acute pancreatitis).

**Table 3:** Multiple linear regression showing the associations between serum uromodulin and eGFR during first 72 h of acute pancreatitis: eGFR was the dependent variable in the models, while age and sex were included as the confounders.

Independent Variables	24 h		48 h		72 h	
	Beta ± SE	*p*	Beta ± SE	*p*	Beta ± SE	*p*
Age	−0.60 ± 0.11	<0.001	−0.57 ± 0.12	<0.001	−0.63 ± 0.12	<0.001
Male sex	−0.03 ± 0.10	NS	0.01 ± 0.11	NS	0.02 ± 0.12	NS
Uromodulin	0.24 ± 0.11	0.037	0.35 ± 0.12	0.006	0.29 ± 0.12	0.020
